# The present approaches to the development of prophylactic and therapeutic antidotes against nerve agents

**DOI:** 10.2478/v10102-010-0026-y

**Published:** 2010-11

**Authors:** Jiří Kassa, Jiří Bajgar, Kamil Kuča, Kamil Musílek, Jana Karasová

**Affiliations:** 1 Department of Toxicology, Faculty of Military Health Sciences, Třebešská 1575, 500 01 Hradec Kralove, Czech Republic; 2 Center of Advanced Studies, Faculty of Military Health Sciences, Třebešská 1575, 500 01 Hradec Kralove, Czech Republic

**Keywords:** nerve agent, prophylaxis, organophosphates, acetylcholinesterase, antidote, therapy

## Introduction

Nerve agents belong to highly toxic organophosphorus compounds misused as chemical warfare agents for military as well as terroristic purposes. Basic mechanism of action of nerve agents is based on acetylcholinesterase (AChE, EC 3.1.1.7) inhibition and subsequent accumulation of neuromediator acetylcholine at the cholinergic synapses, either peripheral or central leading to cholinergic hyperstimulation and development of symptoms of poisoning, followed by metabolic dysbalance and death without effective prophylaxis/treatment. The antidotal treatment of acute poisonings with nerve agents still represents a serious problem and, therefore, we are searching how to satisfactorily protect people against acute toxicity of nerve agents. There are two approaches how to improve the medical protection against nerve agents:

- to increase the resistance of nerve agent-exposed organism and the efficacy of post-exposure antidotal treatment by pharmacological prophylaxis
- the development of new, more effective antidotes, expecially AChE reactivators, to achieve the satisfactorily effective antidotal treatment of acute poisonings with nerve agents.

## Prophylaxis of Nerve Agent Exposure

Prophylaxis against nerve agents is based on various approaches: keeping AChE, key enzyme for toxic action of nerve agents intact (protection of cholinesterases) is a basic requirement for effective prophylaxis. It can be reached using reversible inhibitors (preferably carbamates). AChE inhibited by carbamates is resistant to nerve agent inhibition. After spontaneous recovery of the activity (decarbamylation), normal AChE serves as a source of the active enzyme. Pyridostigmine is the most promising carbamate especially against soman poisoning (Gordon *et al*., 1978; Kassa *et al*., 2001). It was introduced into some armies as a prophylactic drug against nerve agents. Its prophylactic effect (like the effects of other carbamates) is limited by its dose. With a higher dose, a higher efficacy was observed, but the side effects were more expressed, too. This problem can be solved by the adding of pyridostigmine antagonizing drugs – anticholinergics. The prophylactic combination of pyridostigmine with trihexyphenidyle and benactyzine was introduced into the Czech Army as PANPAL. The presence of these two anticholinergics allowed us to increase the pyridostigmine dose and to increase its prophylactic efficacy without side effects as it has been demonstrated on volunteers (Bajgar, 2004; Fusek *et al*., 2006). Other carbamates also have a good prophylactic efficacy, especially physostigmine (due to its central effect on the contrary to pyridostigmine) (Bajgar, 2004; Bajgar *et al*., 2007a; Patocka *et al*., 2006). Structurally different inhibitors from the carbamate and organophosphate groups were also studied. Among these compounds (preferably binding to the AChE anionic site), tacrine, 7-MEOTA and huperzine A were considered and experimentally studied with respect to prophylaxis *in vitro* and *in vivo*. The most interesting results were obtained with huperzine A (Bajgar *et al*., 2007b; Gordon *et al*., 2005; Lallement *et al*., 2002).

Detoxification principle can be used in two different ways: administration of enzymes splitting nerve agents or specific enzymes which bind nerve agents (cholinesterases). Nerve agents are bound to the exogenously administered enzyme and thus the nerve agent level in the organism is decreased (it acts as “scavenger”). Enzymes hydrolysing nerve agents are under research. The administration of enzymes (AChE and butyrylcholinesterase, BuChE, EC 3.1.1.8) as scavengers seems to be very promising. They are acting at the very beginning of the toxic action, without interaction with the target tissues and without side effects. All of these features are of great interest and they are yielding practical results – isolation of the enzyme, examination for lack of and auto immune response, stability, and establishment of pharmacokinetic and pharmacodynamic properties. Moreover, BuChE pretreatment also showed protective effects on AChE inhibition in the brain parts following low level sarin inhalation exposure (Sevelova *et al*., 2004). Given our increasing knowledge in bioengineering and biotechnology, it would be possible to obtain a modified enzyme splitting nerve agents and simultaneously reacting with nerve agents as a scavenger (Bajgar *et al*., 2007c; Doctor *et al*., 1991, 1997; Saxena *et al*., 2004).

The antidotes currently used for the treatment of nerve agent poisoning can be tested as prophylactics. This principle can be considered as a treatment “in advance”. Standard antidotes were studied in this respect i.e. anticholinergics, reactivators, anticonvulsants and others. The problem with their use is the timing, duration and achievement of sufficient levels of these antidotes after the administration. The prolongation of the duration of the antidotal effects by achievement of their sufficient level in the blood by oral administration is not always possible, especially for reactivators. There is a reason for searching for other routes of administration. Transdermal administration of one of the most effective reactivators (HI-6) was shown to be the most realistic approach. The final result was the new prophylactic transdermal antidote TRANSANT containing the reactivator HI-6. This preparation was clinically tested (including dermal sensitivity) without any harmful effects; field testing was also successful and TRANSANT was introduced into the Czech Army (Bajgar, 2004; Fusek *et al*., 2007) .

Combinations of these approaches and use of different drugs are also possible. Benzodiazepines, calcium antagonists, neuromuscular blockers, adamantanes, and the opiate antagonists were tested with different results but they are not very useful for practical use. There are other combinations such as an administration of triesterase, procyclidine, clonidine, sustained release of physostigmine and scopolamine. However, all these studies are just experimental and they have not reached the practical output stage (Bajgar, 2004). Only three prophylactics have been introduced into the armies – PYRIDOSTIGMINE BROMIDE, PANPAL (tablets containing pyridostigmine, trihexyphenidyle and benactyzine), TRANSANT (transdermal patch containing HI-6) are means introduced into different armies as prophylactics at present. It appears from these results that simple prophylaxis (without postexposure treatment) against nerve agents is not sufficient enough. Future development will be focused on scavengers (cholinesterases and modified enzymes) and other drugs – preferably reversible cholinesterase inhibitors (e.g. huperzine A, physostigmine, acridine derivatives etc.).

## The Evaluation of Reactivating Efficacy of New Oximes *In Vitro*

Generally, the design of new pharmaceutically active compounds is based on computational approaches such as docking studies, prediction of active compounds using different predictive approaches or structure-activity relationship. These tools are used for the development of new AChE reactivators, too (Dohnal *et al*., 2005). The most important part influencing further design of new AChE reactivators at our department is the structure-activity relationship study. In this part, we have focused our attention on several structural requirements which are the most important for the sufficient reactivation potency of used oximes. Among them, the presence of the quaternary nitrogen (needed for the affinity to intact and inhibited enzyme), the presence of the oxime group (needed for the breaking of the nerve agent-enzyme complex) and the length of the linker between two pyridinium rings (influencing affinity towards the enzyme) are the most important (Kuca *et al*., 2006).

To evaluate the reactivating efficacy of newly developed and currently available oximes *in vitro*, the brain homogenate (0.5 ml) is treated with solution (0.5 ml) of nerve agent in appropriate concentration for 30 min. Afterwards, reaction solution is adjusted to 23 ml with 0.3 M sodium chloride. Then, 0.02 M solution of acetylcholine iodide (2 ml) is added to the mixture. The liberated acetic acid is titrated with 0.01 M sodium hydroxide on an RTS 822 titrator (Radiometer, Denmark) in the pH-stat mode (pH 8.0) at room temperature (25 °C). The slope of the linear part of the time dependence of the sodium hydroxide used represents the activity of the inhibited enzyme (in fact, the initial rate of the enzymatic reaction). Resulted inhibition of AChE is set to 95%. Reactivation of inhibited AChE is performed immediately after its inhibition. A solution (1 ml) of the reactivator in appropriate concentration is added to the inhibited enzyme. After 10 min reactivation at 25 °C, the mixture is adjusted to 23 ml with 0.3 M sodium chloride solution. Then, 0.02 M solution of acetylcholine iodide (2 ml) is added and immediately afterwards the activity of the reactivated enzyme is determined by above described potentiostatic method (Kuca and Kassa, 2003). The screening test is used as the first step of evaluation of the large quantity of new oximes to reactivate nerve agent-inhibited AChE *in vitro.* As the results, we have obtained a percentage of reactivation. Only promising oximes together with standards are tested in the second step of *in vitro* investigation where their kinetic parameters of reactivation are calculated.

Regarding the structure-activity relationship study, many AChE reactivators were prepared in the last decade. Their design was targeted towards nerve agents (especially tabun) as well as organophosphorus pesticides. In general, the monoquaternary salts (analogues of pralidoxime) were replaced by bisquaternary reactivators (analogues of trimedoxime and obidoxime). Namely, the symmetrical molecules with two oximes were used in effort to extend the reactivation potency (Musilek *et al*., 2006; Musilek *et al*., 2007a). Based on previous results, the further design was adapted for bisquaternary compounds with one oxime and modified non-oxime part of the molecule (Musilek *et al*., 2007b).

The synthesis of new reactivators and the evaluation of their reactivating efficacy *in vitro* resulted in several promising compounds. The first generation is represented by K027 and K048 (Kuca *et al*., 2003), which were found to be effective especially against organophosphorus pesticides and less toxic than most currently available compounds. The second generation of oximes is represented by K074 and K075 (Kuca *et al*., 2005), which extend the reactivation potency of obidoxime and trimedoxime, however, they were highly toxic. Recently, the third generation was developed from all previous results and the best compound against tabun-inhibited AChE was called K203 (Musilek *et al*., 2007c). This compound is well balanced in case of reactivation activity and its toxicity compared to commonly used reactivators. Thus, K203 seems to be very promising oxime among reactivators of tabun-inhibited AChE.

## The Evaluation of Reactivating and Therapeutic Efficacy of New Oximes *In Vivo*

According to in vitro results, the most promising reactivators were subjected to the in vivo studies (toxicity test, the evaluation of therapeutic, reactivating and neuroprotective efficacy) and their potency to counteract the acute toxicity of nerve agents is compared to the therapeutic and reactivating efficacy of commonly used oximes – obidoxime, trimedoxime and the oxime HI-6.

Before starting the evaluation of reactivating and therapeutic efficacy of all newly developed oximes, the acute toxicity of oximes in rats and mice is evaluated by the assessment of the LD_50_ values and their 95% confidence limit using probit-logarithmical analysis of death occurring within 24 hr after i.m. administration of oxime at 5 different doses with 8 animals per dose (Tallarida and Murray, 1987). To evaluate the therapeutic efficacy of newly developed oximes against supralethal poisoning with nerve agent in mice was evaluated by the assessment of the LD_50_ values and their 95% confidence limit using probit-logarithmical analysis of death occurring within 24 hr after i.m. administration of nerve agent at 5 different doses with 8 animals per dose (Tallarida and Murray, 1987). To evaluate their therapeutic efficacy, the oximes were i.m injected at equitoxic doses corresponding to 5% of their LD_50_ value in combination with atropine (21 mg/kg) 1 min after nerve agent administration. The influence of the nature of the antidotal treatment was expressed as protective ratio (LD_50_ value of treated mice/ LD_50_ value of untreated mice).

To evaluate the reactivating efficacy of the oximes, the rats are injected i.m. with either atropine (21 mg/kg) alone or atropine (21 mg/kg) in combination with one of the oximes studied at equitoxic doses corresponding to 5% of their LD_50_ value 5 min before intramuscular nerve agent poisoning in rats. The control rats are administered i.m. with saline instead of nerve agent and antidotes at the same volume. The rats are decapitated and exsanguinated to obtain the blood 30 min following nerve agent poisoning at a dose corresponding to LD_50_. The diaphragms and brains are removed and homogenized in distilled water to determine AChE activity by a spectrophotometric method (Ellman *et al*., 1961). The reactivation rate (%) is calculated using the AChE activity values: {1 – [((saline control) – (oxime + atropine))/((saline control) – (atropine control))]} × 100 (Clement *et al*., 1992). The AChE activity is expressed as µkat/kg or L (µmol substrate hydrolyzed/kg wet tissue or L blood/s).

The development of new oximes is especially directed for the antidotal treatment of acute tabun poisoning because deleterious effects of tabun (*O*-ethyl-*N,N*-dimethylphosphoramido-cyanidate) are extraordinarily difficult to antagonize due to the changes in hydrogen bonding and the conformational changes of AChE-tabun complex after aging process in the AChE active site that make the nucleophilic attack of oxime almost impossible (Ekström *et al*., 2006). According to *in vitro* results, one of the most promising oximes for antidotal treatment of acute tabun poisoning is K203 [1-(4-carbamoylpyridinium)-4-(4-hydroxyiminomethyl-pyridinium)-but-2-ene dibromide]. The results show that the acute toxicity of the newly developed oxime K203 is a little higher than the acute toxicity of obidoxime and trimedoxime in mice but it is significantly lower than the acute toxicity of obidoxime and trimedoxime in rats.

The oxime K203 is able to significantly reactivate tabun-inhibited AChE in blood, diaphragm and brain. Its reactivating efficacy is higher in comparison with the potency of obidoxime and trimedoxime to reactivate tabun-inhibited AChE in diaphragm and brain, but the difference in their reactivating potency is significant in brain only. On the other hand, the oxime HI-6 is considered to be the worst reactivator of tabun-inhibited AChE among currently available oximes (Kassa *et al*., 2008). These results correlate with the therapeutical potency of the oximes tested against lethal tabun poisoning in mice. The potency of newly developed oxime K203 to eliminate lethal toxic effects of tabun in mice was significantly higher in comparison with all commonly used oximes studied. It was able to decrease acute toxicity of tabun nearly two times. On the other hand, the oxime HI-6 showed significantly lower potency to eliminate lethal toxic effects of tabun in mice in comparison with other studied oximes (Kassa *et al*., 2008).

Thus, the newly developed oxime K203 seems to be significantly more efficacious to reactivate tabun-inhibited AChE in rats and to eliminate lethal toxic effects of tabun in mice than all currently available oximes and, therefore, it is suitable for the replacement of commonly used oximes for the treatment of acute tabun poisoning.
